# Epidemiology and Outcomes of Recurrent *C Difficile* Infection Among Hematopoietic Cell Transplant Recipients: A Single-center, Retrospective 10-year Study

**DOI:** 10.1093/ofid/ofae570

**Published:** 2024-10-01

**Authors:** Eduardo Sanchez, Elizabeth M Krantz, Zahra Kassamali Escobar, Frank Tverdek, Emily A Rosen, Masumi Ueda Oshima, Paul A Carpenter, Steven A Pergam, Catherine Liu

**Affiliations:** Division of Infectious Diseases, University of Arizona College of Medicine, Banner University Medical Center-Tucson, Tucson, Arizona, USA; Vaccine and Infectious Disease Division, Fred Hutchinson Cancer Center, Seattle, Washington, USA; Vaccine and Infectious Disease Division, Fred Hutchinson Cancer Center, Seattle, Washington, USA; School of Pharmacy, University of Washington, Seattle, Washington, USA; Vaccine and Infectious Disease Division, Fred Hutchinson Cancer Center, Seattle, Washington, USA; School of Pharmacy, University of Washington, Seattle, Washington, USA; Vaccine and Infectious Disease Division, Fred Hutchinson Cancer Center, Seattle, Washington, USA; Division of Allergy and Infectious Diseases, University of Washington, Seattle, Washington, USA; Clinical Research Division, Fred Hutchinson Cancer Center, Seattle, Washington, USA; Division of Hematology and Oncology, University of Washington School of Medicine, Seattle, Washington, USA; Clinical Research Division, Fred Hutchinson Cancer Center, Seattle, Washington, USA; Department of Pediatrics, University of Washington, Seattle, Washington, USA; Vaccine and Infectious Disease Division, Fred Hutchinson Cancer Center, Seattle, Washington, USA; Division of Allergy and Infectious Diseases, University of Washington, Seattle, Washington, USA; Clinical Research Division, Fred Hutchinson Cancer Center, Seattle, Washington, USA; Vaccine and Infectious Disease Division, Fred Hutchinson Cancer Center, Seattle, Washington, USA; Division of Allergy and Infectious Diseases, University of Washington, Seattle, Washington, USA; Clinical Research Division, Fred Hutchinson Cancer Center, Seattle, Washington, USA

**Keywords:** *C difficile* infection, hematopoietic cell transplant, immunocompromised, recurrent *C difficile* infection, risk factors

## Abstract

**Background:**

There are limited data on the contemporary epidemiology of recurrent *Clostridioides difficile* infection (CDI) among hematopoietic cell transplant (HCT) recipients. We aimed to determine the incidence, risk factors, and outcomes for recurrent CDI among HCT recipients.

**Methods:**

We conducted a retrospective study of adult HCT recipients between 2012 and 2021 diagnosed with index CDI between HCT day −7 and +100. Recurrent CDI was defined as new symptoms and a positive test within 12 weeks after treatment for index CDI. Cox proportional hazards models were used to investigate associations between prespecified variables (age, neutropenia, exposure to antibiotics with antianaerobic coverage, cytomegalovirus viremia/disease, and metronidazole monotherapy) and recurrent infection, presented as hazard ratios with 95% confidence intervals (CI).

**Results:**

Of 3479 HCT recipients, 416 (12%) had index CDI and were treated with oral vancomycin (31%), metronidazole (41%), oral vancomycin and metronidazole (29%). Of 381 patients eligible for recurrent CDI analysis, 35 had recurrent infection; cumulative incidence was 10% (95% CI, 7–13) at 12 weeks. In the 14 days after recurrence, 2/25 (8%) patients required hospital admission; none died within 30 days. Metronidazole monotherapy for treatment of index CDI was associated with an increased rate of recurrence (adjusted hazard ratio, 2.0; 95% CI, 1.0–4.0; *P* = .048).

**Conclusions:**

Recurrent CDI occurred in 10% of HCT recipients in the early posttransplant period and was associated with use of metronidazole. Further study is needed to characterize risk factors for recurrent CDI among HCT recipients to guide use of agents aimed at preventing recurrence.

## Teaser

Recurrent CDI occurred in 10% of HCT recipients in the early posttransplant period without deaths or severe complications. Metronidazole use for treatment of the initial episode of CDI was associated with an increased rate of recurrent infection.

*Clostridioides difficile* infection (CDI) is a leading cause of hospital-acquired infections among hematopoietic cell transplant (HCT) recipients [1, 2]. Several factors predispose these individuals to CDI, including prolonged hospitalization, disruption of the normal gut microbiota through frequent antibiotic exposure and receipt of cytotoxic conditioning chemotherapy, and impaired immune response to *C difficile* toxins [3–5]. Retrospective studies demonstrate that the incidence of CDI following HCT ranges from 5% to 20% with variability in part from differences in transplant populations and *C difficile* testing strategies [6–12].

The propensity for recurrent infection following initial CDI is a challenge to management and in this vulnerable population, may contribute to increased morbidity [13]. Retrospective studies have reported recurrence rates ranging from 11% to 33% [3, 14, 15], but there are few studies that have investigated the clinical outcomes and risk factors for recurrent CDI in HCT recipients [3, 10, 16]. A contemporary understanding of the burden of and risk factors associated with recurrent CDI in HCT recipients is needed to guide use of therapies intended to limit recurrent CDI such as fidaxomicin and bezlotoxumab [17, 18].

To characterize the epidemiology of recurrent CDI at our transplant center, we aimed to first determine the prevalence of index CDI and the incidence of recurrent CDI, then, as secondary aims, to characterize and describe clinical outcomes as well as risk factors associated with recurrent CDI among HCT recipients.

## METHODS

### Study Design

We performed a single-center retrospective cohort study of adults age ≥18 years who received their first autologous or allogeneic HCT at the Fred Hutchinson Cancer Center (FHCC) between 1 January 2012 and 31 December 2021 and who developed CDI between day −7 and day +100 relative to transplant. The study population was identified using an electronic center database of HCT recipients. We extracted patient demographic and clinical characteristics and captured, through individual chart review, the outcomes measured up to 14 days after diagnosis of the initial and recurrent CDI episode including CDI-associated hospitalization and intensive care unit (ICU) admission, surgical intervention, and complications including toxic megacolon, ileus, and shock; mortality was evaluated at 30 days following CDI diagnosis.

## PATIENT CONSENT STATEMENT

This study was approved by the FHCC institutional review board and was exempt from informed consent.

### Definitions

CDI was defined by the presence of diarrhea (3 or more unformed stools in 24 hours) and a positive test. During the study period, only polymerase chain reaction (Cepheid, Sunnyvale, CA) testing was available; no toxin testing was performed. We defined an index CDI episode as the first CDI episode occurring between day –7 and day +100 relative to HCT for each patient. Recurrent CDI was defined as a new episode of diarrhea with a positive CDI test within 12 weeks after completing primary treatment for the index CDI. Treatment with metronidazole was standard practice at our center until 2018, after which oral vancomycin was primarily used. A treatment course for the index CDI was determined using prescription data, including records of consecutive orders or administrations of oral vancomycin, metronidazole, or fidaxomicin starting at the date of the CDI diagnosis and allowing for a gap of up to 7 days between order dates to account for outpatient prescriptions following hospital discharge. Because of limitations with prescription data, treatment courses that were <14 days by this method were reassigned a treatment duration of 14 days, unless the patient died or was lost to follow-up before day 14.

We defined the following antibiotics as those with antianaerobic coverage: amoxicillin/clavulanate, ampicillin/sulbactam, clindamycin, ertapenem, imipenem-cilastatin, doripenem, meropenem, piperacillin/tazobactam, metronidazole, or moxifloxacin. Cytomegalovirus (CMV) viremia or disease was defined by the presence of detectable CMV viral load requiring treatment or evidence of end-organ damage associated with CMV in the week before initial CDI diagnosis. Neutropenia was defined as an absolute neutrophil count <500 cells/μL within 48 hours before CDI diagnosis. CDI was considered hospital-acquired if the diagnosis was made after day 3 of hospitalization. Last date of FHCC care was defined by 2 weeks after the last documented date patients received care at FHCC, from date of treatment completion for the index CDI episode to 6 months later.

### Statistical Analysis

To describe the prevalence of CDI and characterize and describe clinical outcomes for patients with CDI, we included all patients with an index CDI who received treatment. To compute prevalence, we used a denominator of all patients aged ≥18 years who received their first HCT at FHCC between 1 January 2012 and 31 December 2021. Data were summarized using descriptive statistics, including the median and range for continuous variables and frequency tabulations and percentages for categorical variables.

To evaluate the cumulative incidence of and risk factors for recurrent CDI, we defined time zero as date of treatment completion for the index CDI episode. Recurrent CDI was evaluated up until the earliest of the following: last date of FHCC care, death date, 12 weeks after treatment completion of the index CDI. For this analysis, we excluded patients with <1 day of follow-up and those missing data for key risk factors (see the following section) we considered for recurrence. We also excluded those with a CDI in the 98 days before the index CDI. Because our definition of recurrent CDI used a 12-week (84 days) follow-up period after completion of treatment (which is generally 14 days), we used the same period (98 days) before the index episode to exclude index CDI episodes that could potentially be recurrences and not initial episodes of CDI.

Cumulative incidence of recurrent CDI was computed with 95% confidence intervals (CI), treating death as a competing risk. Cox proportional hazards univariable and multivariable models were used to investigate associations between prespecified variables previously shown to be risk factors [3, 10, 16, 19] (age at HCT, neutropenia at index CDI, use of antibiotics with antianaerobic coverage in days −7 to +14 relative to index CDI, CMV viremia or disease in week before index CDI, metronidazole monotherapy for treatment of index CDI) for recurrent CDI. All prespecified variables were included in the multivariable model. Model estimates were presented as hazard ratios (HR) with 95% CIs.

We performed a separate analysis of patients excluded from the recurrent CDI analyses because of a CDI episode in the 98 days before the index CDI episode and computed cumulative incidence in this subgroup. We also performed 2 sensitivity analyses for the cumulative incidence of recurrent CDI; first, we excluded patients who received ≥17 days of treatment for the index CDI and second, we allowed a prescription dates gap of up to 3 days rather than 7 days to define treatment course for the index CDI. Further details describing these additional analyses are included in the supplemental materials. SAS, version 9.4 (SAS Institute, Cary, NC), and Stata, version 17 (StataCorp, College Station, TX), were used for all analyses.

## RESULTS

### Index CDI Prevalence and Patient Characteristics

**Figure 1.**
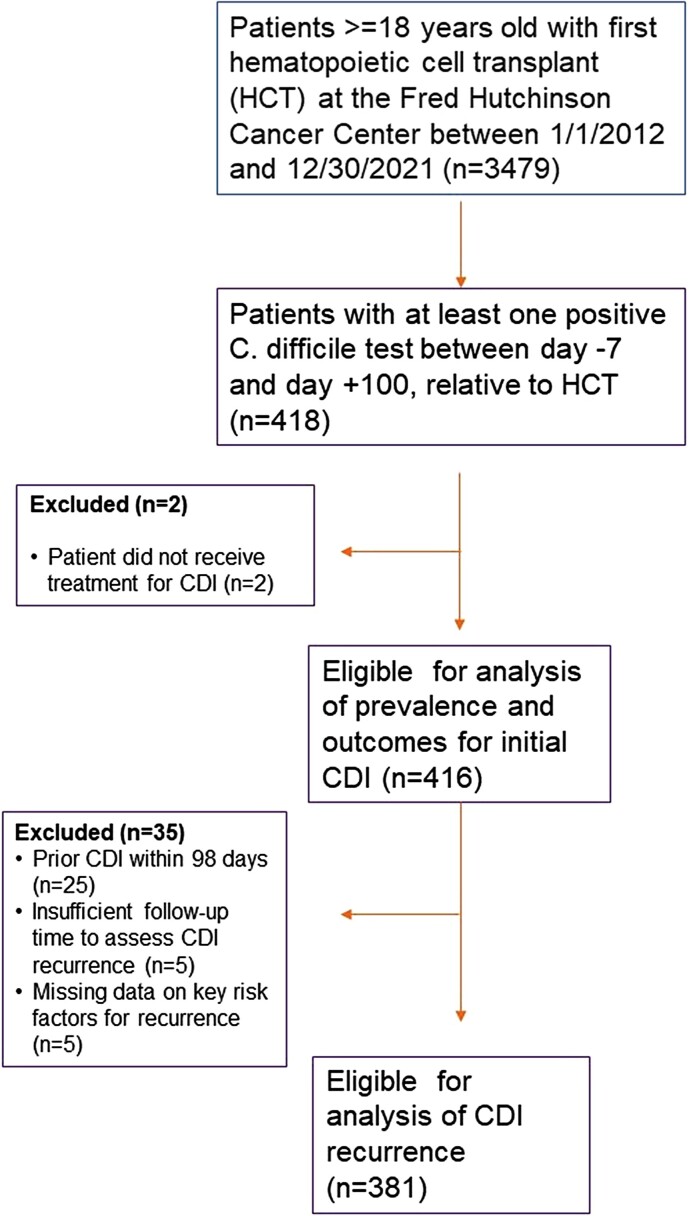
Study flowchart.

Among 3479 HCT recipients during the study period, 416 (12%) patients were diagnosed and treated for an index episode of CDI between day –7 and day +100 relative to HCT (Figure 1). One hundred and twenty-seven patients (30.5%) were treated with oral vancomycin monotherapy, 169 patients (41%) were treated with metronidazole monotherapy, 119 (29%) received both oral vancomycin and metronidazole, and 1 (0.2%) patient received fidaxomicin. The median time from transplant to index CDI was 5 days (range, −7–97). Table 1 describes baseline patient characteristics for those with an index CDI episode. Two hundred and thirty-eight (57%) patients were male and the median age at HCT was 56 years (range, 19–80). Two hundred and forty-eight (60%) underwent allogeneic HCT. Thirty-six (9%) patients had a history of CDI in the year before their index case. Sixty-four (15%) patients were exposed to antibiotics with anaerobic coverage from day −7 to day 14 relative to the index CDI episode.

**Table 1.** Baseline Patient Characteristics of HCT Patients With *C Difficile* Infection^a^

Characteristic Demographics and Clinical Characteristics	Allogeneic HCT (n = 248)	Autologous HCT (n = 168)	All (n = 416)
Age at HCT in years, median (range)	53 (20–80)	59 (19–74)	56 (19–80)
Sex			
Female	107 (43%)	71 (42%)	178 (43%)
Male	141 (57%)	97 (58%)	238 (57%)
Underlying malignancy			
Acute leukemia	176 (71%)	1 (0.6%)	177 (43%)
Chronic leukemia	10 (4%)	0 (0%)	10 (2%)
Hodgkin lymphoma	2 (0.8%)	11 (7%)	13 (3%)
Non-Hodgkin lymphoma	4 (2%)	50 (30%)	54 (13%)
Myelodysplastic syndrome	47 (19%)	0 (0%)	47 (11%)
Multiple myeloma	1 (0.4%)	93 (55%)	94 (23%)
Other	8 (3%)	13 (8%)	21 (5%)
Stem cell source			
Peripheral blood	199 (80.2%)	168 (100%)	367 (88.2%)
Cord	35 (14.1%)	0 (0%)	35 (8.4%)
Bone marrow	13 (5.2%)	0 (0%)	13 (3.1%)
Bone marrow, peripheral blood	1 (0.4%)	0 (0%)	1 (0.2%)
Location of HCT			
Inpatient	216 (87%)	109 (65%)	325 (78%)
Outpatient	32 (13%)	59 (35%)	91 (22%)
Donor relationship			
Autologous	0 (0%)	168 (100%)	168 (40%)
Matched	176 (71%)	–	176 (42%)
Haploidentical	16 (6.5%)	–	16 (4%)
Mismatched	21 (8.5%)	–	21 (5%)
Cord blood	35 (14%)	–	35 (8%)
Presence of acute GVHD^b^	34 (14%)	–	34 (8%)
Acute GVHD grade^c^			
1	1 (3%)	–	1 (3%)
2	27 (79%)	–	27 (79%)
3	5 (15%)	–	5 (15%)
4	1 (3%)	–	1 (3%)
Prior history of CDI^d^	19 (8%)	17 (10%)	36 (9%)
Exposure to proton pump inhibitors^e^	129 (52%)	93 (55%)	222 (54%)
Positive CMV serostatus at transplant	148 (61%)	102 (61%)	250 (61%)
Diagnosis of CMV infection^f^	13 (5%)	1 (1%)	14 (3%)
Engraftment at CDI diagnosis^g^	65 (26%)	41 (24%)	106 (25%)
Concomitant infection at CDI diagnosis	64 (26%)	18 (11%)	82 (20%)
Exposure to antibiotics with anaerobic coverage	37 (15%)	27 (16%)	64 (15%)
Initial CDI episode characteristics			
Days from transplant to CDI median (range)	3.5 (−7 to 97)	6 (−5 to 93)	5 (−7 to 97)
Fever^h^	35 (15%)	52 (33%)	87 (22%)
WBC count cell/microliter median (range)^i^	1495 (0–19,700)	1060 (0–15,270)	1245 (0–19,700)
Neutropenia^j^	84 (34%)	77 (46%)	161 (39%)
Treatment regimens used for index CDI			
Oral vancomycin	79 (31.9%)	48 (28.6%)	127 (30.5%)
Oral metronidazole	61 (24.6%)	45 (26.8%)	106 (25.5%)
Oral and IV metronidazole	25 (10%)	28 (16.7%)	53 (12.8%)
IV metronidazole	7 (2.8%)	3 (1.8%)	10 (2.4%)
Oral vancomycin + metronidazole (IV or oral)	75 (30%)	44 (26.1%)	119 (28.6)
Fidaxomicin	1 (0.4%)	0 (0)	1 (0.2%)
Duration of treatment regimen for index CDI, median days (range)	14 (4–34)	14 (6–38)	14 (4–38)

Abbreviations: CDI, *Clostridioides difficile* infection; CMV, cytomegalovirus; GVHD, graft versus host disease; HCT, hematopoietic cell transplant; WBC, white blood cell.

^a^Among n = 416 total patients. Numbers shown are n (%) unless otherwise specified. Numbers may not add to totals because of missing data, which composed <1% of the cohort for all variables except for fever, which was missing for 28 patients (7%).

^b^Presence of acute GVHD before or at initial CDI diagnosis.

^c^Percentages computed among those with acute GVHD.

^d^History of CDI within the last year before index CDI.

^e^Exposure to proton pump inhibitors in the week before initial CDI diagnosis.

^f^Diagnosis of CMV infection/disease in the week before initial CDI diagnosis.

^g^Engraftment defined as 3 consecutive days of an absolute neutrophil count ≥500 cells/μL.

^h^Temperature ≥38 degrees Celsius within 24 hours before or after CDI diagnosis.

^i^Nearest measurements before or at CDI diagnosis.

^j^Within 48 hours before CDI diagnosis.

Fourteen (3%) patients had a diagnosis of CMV viremia/disease in the week before their CDI index case. All except 1 patient had received an allogeneic HCT and 4 patients had concomitant gastrointestinal acute graft versus host disease (GVHD).

### Clinical Outcomes of Index CDI

Of the 416 patients with index CDI, 10 (2%) died within 30 days of CDI diagnosis; however, none of the deaths was attributable to CDI. Among 119 outpatients at diagnosis of index CDI, 38 (32%) required hospitalization for their CDI. Severe complications were only present in 1 patient who was diagnosed with shock (Table 2).

**Table 2.** Outcomes of Initial and Recurrent *C Difficile* Infection Episodes

Outcome	Initial CDI Episode^a^	Recurrent CDI^b^
Location of patient at CDI diagnosis		
Inpatient	293/412 (71%)	10/35 (29%)
Outpatient	119/412 (29%)	25/35 (71%)
Hospitalization associated with CDI^c^	38/119 (32%)	2/25 (8%)
Days to hospitalization^d^, median (range)	2 (0–14)	4 (0–7)
Surgical intervention	0/412 (0%)	0/35 (0%)
ICU admission associated with CDI^e^		
Yes	3/410 (0.7%)	0/34 (0%)
Days to ICU admission^f^, median (range)	8 (7–8)	–
Severe complications	1/412 (0.2%)	0/35 (0%)
Toxic megacolon	0/412 (0%)	0/35 (0%)
Ileus	0/412 (0%)	0/35 (0%)
Shock	1/412 (0.2%)	0/35 (0%)
Days to shock, median (range)	8	–
Death within 30 d	10/416 (2%)	0/35 (0%)
Days to death, median (range)	18 (3–30)	–

Abbreviations: CDI, *Clostridioides difficile* infection; ICU, intensive care unit.

^a^Among n = 416 total patients. Numbers shown are n (%) unless otherwise specified. For summaries of continuous variables among groups of only a single patient, the value is shown rather than the median and range. Numbers may not add to totals because of missing data. Outcomes were evaluated at 14 days after CDI diagnosis unless otherwise stated. Nonmortality outcomes could not be ascertained for 4 patients.

^b^Among n = 381 total patients eligible for recurrence analysis. Numbers shown are n (%) unless otherwise specified. For summaries of continuous variables among groups of only a single patient, the value is shown rather than the median and range. Numbers may not add to totals because of missing data. Outcomes were evaluated at 14 days after CDI diagnosis unless otherwise stated.

^c^Among patients who were in the outpatient setting at diagnosis for their first CDI episode or recurrence.

^d^Among patients diagnosed in the outpatient setting who became hospitalized.

^e^Among n = 410 patients not already in the ICU at the time of initial CDI diagnosis and among n = 34 not already in the ICU for recurrent CDI.

^f^Among patients not already in the ICU at diagnosis, who were admitted to the ICU.

### Cumulative Incidence of and Risk Factors for Recurrent CDI

**Figure 2.**
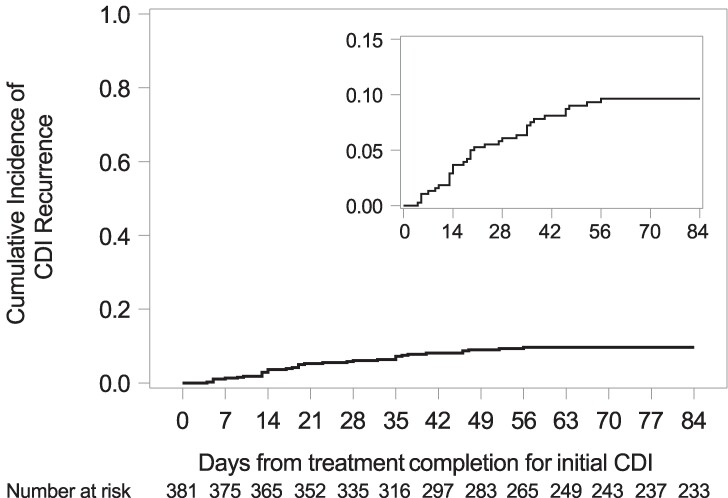
Cumulative incidence of recurrent CDI. Among the 381 patients, 35 had recurrent CDI and 18 had a competing risk death. The cumulative incidence of recurrent CDI at 12 weeks after treatment completion for the index CDI was 10%, 95% confidence interval, 7–13. CDI, *Clostridioides difficile* infection.

Among the 416 patients included in the analysis of prevalence and outcomes for index CDI, 381 were eligible for analysis of CDI recurrence (Figure 1). Median follow-up time for assessing recurrent CDI was 84 days (range, 1–84; interquartile range, 61–84 days). Thirty-five of 381 patients eligible for analysis had recurrent CDI. The cumulative incidence of recurrent CDI at 12 weeks following treatment completion was 10%, 95% CI (7–13) (Figure 2), and did not differ significantly by type of HCT (Supplementary Figure 1). The use of metronidazole monotherapy compared to oral vancomycin with or without metronidazole for the treatment of the index CDI episode was significantly associated with an increased rate of recurrent CDI in the multivariable analysis (HR, 2.0; 95% CI, 1.0–4.0; *P* = .048; Table 3). Age, neutropenia at index CDI, CMV viremia/disease in the week before index CDI, and concurrent use of antibiotics with antianaerobic coverage were not significantly associated with rate of recurrent CDI.

**Table 3.** Cox Model Estimates for Associations Between Clinical Variables and Recurrent *C Difficile* Infection

			Univariable Models		Multivariable Model^a^	
Covariate	N (%)	Cumulative Incidence of Recurrent CDI at 12 wk (95% CI)	Unadjusted HR (95% CI)	*P* Value	Adjusted HR (95% CI)	*P* Value
Age at transplant						
<65 y	287 (75%)	11% (8–15)	1.0 (Reference)		1.0 (Reference)	
≥65 y	94 (25%)	4% (1–10)	0.4 (0.1–1.1)	.08	0.4 (0.1–1.2)	.10
Neutropenia^b^						
No	236 (62%)	9% (6–13)	1.0 (Reference)		1.0 (Reference)	
Yes	145 (38%)	11% (6–16)	1.2 (0.6–2.4)	.57	1.3 (0.7–2.7)	.40
Use of antibiotics with antianaerobic coverage^c^						
No	325 (85%)	10% (7–13)	1.0 (Reference)		1.0 (Reference)	
Yes	56 (15%)	9% (3–19)	1.0 (0.4–2.5)	.96	1.1 (0.4–2.8)	.90
CMV viremia or disease^d^						
No	368 (97%)	9% (6–125)	1.0 (Reference)		1.0 (Reference)	
Yes	13 (3%)	24% (5–50)	2.8 (0.8–9.0)	.09	3.4 (1.0–11.6)	.051
Treatment for the initial CDI						
Oral vancomycin +/− metronidazole	222 (58%)	7% (4–11)	1.0 (Reference)		1.0 (Reference)	
Metronidazole monotherapy	159 (42%)	13% (8–19)	1.9 (0.9–3.6)	.07	2.0 (1.0–4.0)	.048

Abbreviations: CDI, C*lostridioides difficile* infection; CI, confidence interval; CMV, cytomegalovirus; HR, hazard ratio.

^a^Adjusted for age, neutropenia, use of antibiotics with antianaerobic coverage, CMV viremia or disease, and metronidazole monotherapy for treatment of the initial CDI.

^b^Absolute neutrophil count <500 cells/μL at the time of CDI diagnosis.

^c^Use of 1 of the following antibiotics between day −7 and day +14 relative to initial CDI diagnosis: amoxicillin/clavulanate, ampicillin/sulbactam, clindamycin, ertapenem, imipenem, meropenem, doripenem, piperacillin/tazobactam, metronidazole, moxifloxacin. Excludes antibiotics used to treat initial CDI.

^d^Presence of detectable CMV viral load requiring treatment or evidence of end-organ damage associated to CMV between in the week before initial CDI diagnosis.

### Clinical Outcomes of Recurrent CDI

Of the 35 patients with recurrent CDI, none died or had severe complications (Table 2). Ten patients were already in the hospital at the time of the recurrent CDI diagnosis and, based on chart review, CDI was not determined to have prolonged their hospitalization in all cases but 1. Among the 25 who were outpatient at diagnosis, 2 (8%) were hospitalized because of their infection. None of the patients were admitted to the ICU for the recurrent CDI.

### Sensitivity Analyses

Among 23 patients who were excluded from the recurrent CDI analysis because of a CDI episode in the 98 days before the index CDI episode, 4 had a recurrent CDI, for a cumulative incidence of 18% (95% CI, 5–37) at 12 weeks following treatment completion (Supplementary Figure 2). In analyses excluding patients with a treatment course of ≥17 days for the initial CDI episode or requiring shorter gaps in prescription data to define initial treatment course, the cumulative incidence of recurrent CDI was very similar to the original analysis.

## DISCUSSION

In this cohort of HCT patients diagnosed with CDI between day –7 and day + 100 over a 10-year period, the cumulative incidence of recurrent infection was 10% in the 12 weeks after treatment completion for the index episode. There were few hospitalizations, serious complications, or deaths associated with recurrent CDI. We observed a significantly higher rate of recurrent CDI among patients who received metronidazole monotherapy as treatment for their index CDI.

Although the prevalence of index CDI of 12% observed in our study is similar to other studies [14], the rate of recurrent CDI is lower than that previously described. There is significant variability in published rates of recurrent CDI in HCT recipients ranging from 11% to 33% [3, 14, 15], likely reflecting differences in testing approaches as well as the time interval during which recurrence was measured. Potential explanations for our lower observed rates of recurrent CDI include use of *C difficile* polymerase chain reaction as the primary test modality during the study period. It is possible that some of our index episodes of CDI represented colonization rather than true CDI in a population with a high prevalence of diarrhea. Additionally, our recurrent CDI rate at 12 weeks might be expected to be lower than estimates from other studies that measured recurrent CDI up to 1 year posttransplant, when there could be greater opportunity to observe recurrent CDI, simply because of a potentially longer period of observation.

In contrast to other studies suggesting an increased risk of death among patients with recurrent CDI [20, 21], we did not observe any deaths, ICU admissions, or severe complications among patients with recurrent CDI. This finding could be due to the close follow-up and access to care in our patient population, which may have led to early diagnosis and treatment. Of note, our study also showed favorable outcomes after index CDI, including low rates of ICU admission, severe complications, and surgical interventions.

Our findings support existing guidelines that metronidazole should not be used as initial therapy for CDI [18, 22]. In our study of HCT recipients, the use of metronidazole monotherapy for the treatment of index CDI was associated with a higher rate of recurrent CDI. It is unclear whether this association is attributable to a difference in efficacy between metronidazole and other agents used for the treatment of CDI. There are conflicting data regarding the association between metronidazole therapy and the risk of recurrent infection. Several studies, in both the general population and immunocompromised patients, have not shown a significant difference in CDI recurrence rates in patients treated with metronidazole compared to vancomycin [23–27]. However, recent studies that included both adult and pediatric patients showed an increased risk of recurrence in patients treated with metronidazole compared with oral vancomycin [28–32], similar to the findings in our study. This may be related to metronidazole's inferiority in achieving clinical cure compared to vancomycin [33, 34] as well as the decrease in metronidazole stool levels once colonic inflammation subsides, which could lead to a decreased microbiologic response [35, 36].

Updated guidelines by the Infectious Diseases Society of America and the American Society for Transplantation and Cellular Therapy preferentially recommend using fidaxomicin as first-line treatment for CDI [17, 18] to reduce risk of recurrent CDI. In randomized controlled trials of adult non-HCT patients, fidaxomicin was associated with lower rates of recurrent CDI when compared to oral vancomycin [37, 38] and less disruption to the microbiome [39]. During our study period, only 1 patient received fidaxomicin and thus we were unable to evaluate the effect of fidaxomicin on recurrent CDI. However, it is notable that the rate of recurrent CDI in our cohort was substantially lower than the 25%–27% 4-week recurrence rates observed among patients receiving oral vancomycin in the Food and Drug Administration licensing trials for fidaxomicin [37, 38].

Identification of HCT recipients at highest risk for recurrent CDI may help inform targeted use of therapies such as fidaxomicin and bezlotoxumab to populations most likely to benefit, particularly in centers where local epidemiology indicates relatively low rates of recurrence. In contrast to other studies [40, 41], we did not observe an association between older age and recurrent CDI. Limited data from prior studies in HCT recipients have identified gastrointestinal GVHD, antibiotic exposure before CDI, CMV viremia, and neutropenia at the time of diagnosis as risk factors for recurrent CDI in the HCT population [3, 7, 10, 16]. We did not find associations between these factors and recurrent CDI, though we were not able to consider GVHD. In the general population, history of CDI in the past 6 months has been linked to higher rates of recurrent CDI [42]. Although we excluded those patients with a recent history of CDI from our primary analysis, which focused on index CDI, the cumulative incidence of recurrent CDI was 18% in this group, higher than what was observed among those with index CDI. Though beyond the scope of this study, additional investigation is needed to characterize those who may be at risk for multiple episodes of CDI to guide approaches to prevention.

Our study had several limitations, including its retrospective design, which is subject to intrinsic sources of bias and confounding. It is possible that we may have underestimated the incidence of recurrent CDI because of a lack of capture of episodes outside our system; however, we believe this is less likely from the close follow up during the early posttransplant period. Because we are unable to quantify the impact of *C difficile* colonization on the rate of index CDI, the incidence of recurrent CDI may be an underestimate if the true rate of index CDI is lower than described. Given the low rate of recurrent CDI, it is possible that the study was underpowered to detect significant associations with our included variables, and we were unable to evaluate additional potential predictor variables. We were limited in our ability to collect details on antibiotic exposures before the index CDI episode including whether or not patients were compliant with their prescriptions for the index episode. However, in sensitivity analyses that explored different definitions for treatment courses for the index CDI or excluded patients with very long treatment courses, we found very similar rates of recurrent CDI. We were also not able to evaluate the effect of any changes in infection prevention practices or diagnostic stewardship interventions targeting CDI on infection rates. Because of the inclusion of both allogeneic and autologous HCT recipients in our cohort with an overall small number of patients with GVHD, we were unable to evaluate the impact of CDI on GVHD outcomes or the effect of GVHD on the risk of recurrent CDI. Because disruption of the gut microbiota is associated with risk of GVHD, further studies are needed to assess the impact of CDI and recurrent CDI on GVHD outcomes.

## CONCLUSION

We observed relatively low rates of recurrent CDI with no deaths or severe complications among our cohort of HCT recipients. The rate of recurrent CDI was significantly higher in patients who received metronidazole monotherapy for the treatment of their index episode. Further study is needed to determine additional risk factors for recurrent CDI among HCT recipients that may guide use of therapies aimed to reduce recurrence to patients most likely to benefit.

## Supplementary Material

ofae570_Supplementary_Data

## Supplementary Data

Supplementary materials are available at *Open Forum Infectious Diseases* online. Consisting of data provided by the authors to benefit the reader, the posted materials are not copyedited and are the sole responsibility of the authors, so questions or comments should be addressed to the corresponding author.

## Notes

***Author contributions.*** E.S.: study design, data collection and analyses, interpretation of results, manuscript writing; E.M.K.: study design, data analyses, critical review, manuscript writing; S.A.P.: study design, interpretation of results, critical review; and C.L.: study design and supervision, data collection, interpretation of results, critical review and manuscript writing. All other authors contributed to critical review of the manuscript.

***Financial support*.** Research was supported by the National Institutes of Health/National Institute of Allergy and Infectious Diseases under Award Number T32AI118690 and National Institutes of Health/National Cancer Institute Grants P30 CA15704.
