# Supplementary material for: Epidemiology and Outcomes of Recurrent C Difficile Infection Among Hematopoietic Cell Transplant Recipients: A Single-center, Retrospective 10-year Study
Source: Open Forum Infect Dis. 2024 Oct 1;11(10):ofae570. doi: 10.1093/ofid/ofae570 (PMC11500450; doi:10.1093/ofid/ofae570)
Supplement: ofae570_Supplementary_Data [file ofae570_supplementary_data.docx]

**Supplemental materials**

**METHODS**: We performed a separate analysis of patients excluded from the recurrent CDI analyses due to a CDI episode in the 98 days before the index CDI episode and computed cumulative incidence in this subgroup. We also performed two sensitivity analyses that estimated the cumulative incidence of recurrent CDI in a different subgroup of patients or using different methods than our main analysis. The first analysis excluded patients who received ≥17 days of treatment for the index CDI to assess the impact of patients who either remained on secondary prophylaxis for CDI in the setting of continued antibiotic therapy or those who were on an oral vancomycin taper. We selected 17 days of treatment as our cutoff by extending the longest standard course of 14 days by a small margin of error to account for weekend gaps in prescriptions. The second sensitivity analysis allowed a gap of up to 3 days in prescription dates instead of 7 days to define treatment course for the index CDI. Gaps in prescription dates were included to account for the transition period between the inpatient and outpatient setting. This analysis was performed to examine whether our method used to define treatment course for the index CDI, and thus define time zero for our recurrent CDI analysis, influenced our estimates of recurrent CDI. We chose to examine a shorter gap time for the sensitivity analysis to evaluate whether a stricter definition of treatment course would result in different estimates of recurrent CDI.

**RESULTS:** There was no significant difference in the cumulative incidence of recurrent CDI between allogeneic and autologous HCT (Supplemental Figure 1). The cumulative incidence of recurrent CDI among 23 patients who were excluded from the recurrent CDI analysis due to a CDI episode in the 98 days before the index CDI episode is shown in Supplemental Figure 2; cumulative incidence was 18% (95% CI 5%-37%) at 12 weeks following treatment completion. In sensitivity analyses excluding patients with a treatment course of ≥17 days for the initial CDI episode, the cumulative incidence of recurrent CDI at 12 weeks (10%, 95% CI 7%-14%) was nearly identical to the estimate in original analysis. In sensitivity analyses requiring shorter gaps in prescription data to define initial treatment course, the cumulative incidence of recurrent CDI at 12 weeks was the same as in the original analysis.

Supplemental Figure 1. Cumulative incidence of CDI recurrence by type of HCT (allogeneic versus autologous). Numbers shown below the x-axis represent the number of patients at risk in each group.

Supplemental Figure 2. Cumulative incidence of CDI recurrence among 23 patients who were excluded from the recurrent CDI analysis due to a CDI episode in the 98 days before the index CDI episode. There were 4 patients with recurrent CDI and 1 patient with a competing risk death.
